# PCSK9 Inhibitors: The Evolving Future

**DOI:** 10.1002/hsr2.70174

**Published:** 2024-10-30

**Authors:** Bijay Mukesh Jeswani, Shubhangi Sharma, Sawai Singh Rathore, Abubakar Nazir, Rohit Bhatheja, Kapil Kapoor

**Affiliations:** ^1^ Department of Medicine GCS Medical College, Hospital & Research Centre Ahmedabad India; ^2^ Department of Medicine B.J. Governement Medical College Pune India; ^3^ Department of Medicine Dr. Sampurnanand Medical College Jodhpur Rajasthan India; ^4^ Department of Medicine King Edward Medical University Lahore Pakistan; ^5^ Department of Medicine Oli Health Magazine Organization, Research, and Education Kigali Rwanda; ^6^ Cardiology, AdventHealth Orlando Orlando Florida USA

**Keywords:** cholesterol cardiovascular disease, familial hypercholesterolemia, PCSK9 inhibitors LDL, proprotein convertase, subtilisin/kexin type 9

## Abstract

**Introduction:**

PCSK9 inhibitors are a novel class of medications that lower LDL cholesterol (LDL‐C) by increasing LDL receptor activity, promoting clearance of LDL‐C from the bloodstream. Over the years, PCSK9 inhibitors have been explored as adjunct therapies to statins or as monotherapy in high‐risk cardiovascular patients.

**Aim:**

This review aims to provide an updated perspective on PCSK9 inhibitors, assessing their clinical efficacy, safety, and significance, especially in light of recent clinical trials.

**Methods:**

The review examines the role of PCSK9 in cholesterol regulation and summarizes the results of major cardiovascular trials, including FOURIER, SPIRE‐1, SPIRE‐2, and ODYSSEY Outcomes. It also discusses emerging treatments like small interfering RNA (siRNA) therapies and evaluates PCSK9 inhibitor effects on LDL‐C and lipoprotein(a) levels.

**Results:**

Clinical trials have shown PCSK9 inhibitors reduce LDL‐C by up to 60%. In the FOURIER trial, evolocumab reduced LDL‐C by 59% and major cardiovascular events by 15%–20%. The SPIRE‐2 trial, despite early termination, showed a 21% risk reduction in the primary composite endpoint with bococizumab. The ODYSSEY Outcomes trial reported a 57% LDL‐C reduction with alirocumab, alongside a 15% reduction in adverse events. Emerging treatments like Inclisiran offer long‐term LDL‐C control with fewer doses. PCSK9 inhibitors are generally well‐tolerated, with the most common side effect being injection site reactions.

**Conclusion:**

PCSK9 inhibitors significantly lower LDL‐C and reduce cardiovascular events, offering promising therapies for high‐risk patients, including those with familial hypercholesterolemia (FH) and those who cannot tolerate statins. Future research will focus on optimizing these inhibitors, integrating complementary therapies, and exploring gene‐editing technologies to improve patient outcomes.

## 1 Introduction to PCSK9 Inhibitors

**Figure 1:**
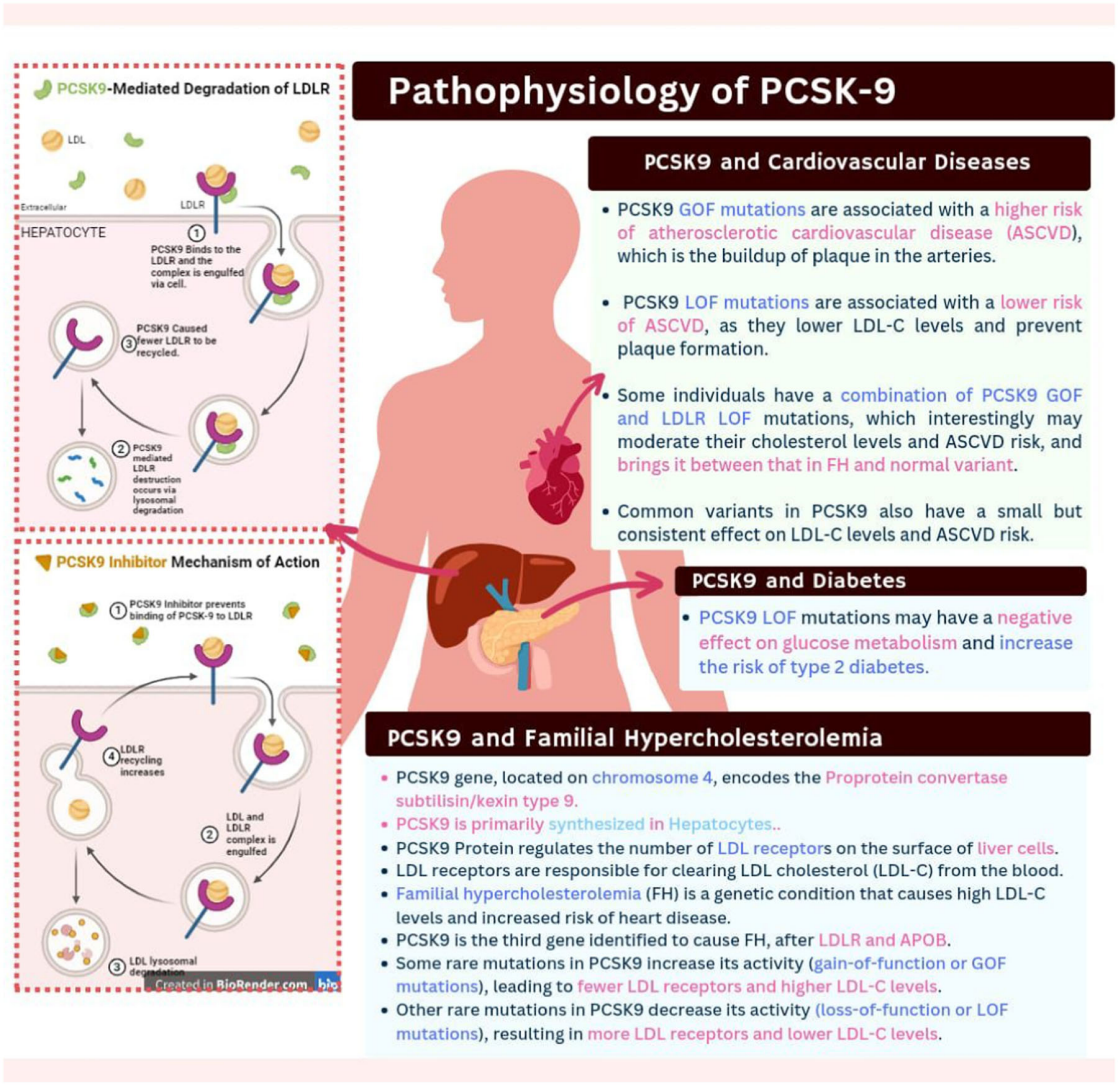
Role of PCSK9 in LDL receptor regulation.

The history of PCSK9 inhibition begins with pioneering genetic discoveries that clarified PCSK9's function in controlling LDL cholesterol (LDL‐C) levels and its effects on cardiovascular health [1]. In the absence of a mutation in the gene encoding LDL receptor or apolipoprotein B (APOB), certain families were found with the diagnosis of familial hypercholesterolemia (FH), an autosomal codominant genetic disorder characterized by elevated levels of LDL‐C and early atherosclerosis [2]. Subsequent linkage analyses and classic Mendelian genetics identified a region on chromosome 1 that co‐segregated with this FH phenotype [3]. Mutations in this gene underlie autosomal dominant hypercholesterolemia, and subsequent candidate gene studies delineated the PCSK9 protein's indispensable role in the life cycle of LDL receptors, responsible for the clearance of LDL particles from the circulation [4]. Hepatocytes secrete PCSK9, which binds to the LDL receptor, preventing it from recirculating to the cell surface. This leads to increased numbers of LDL receptors being degraded and reduced amounts of LDL‐C being cleared from the bloodstream by the liver (Figure 1).

**Figure 2:**
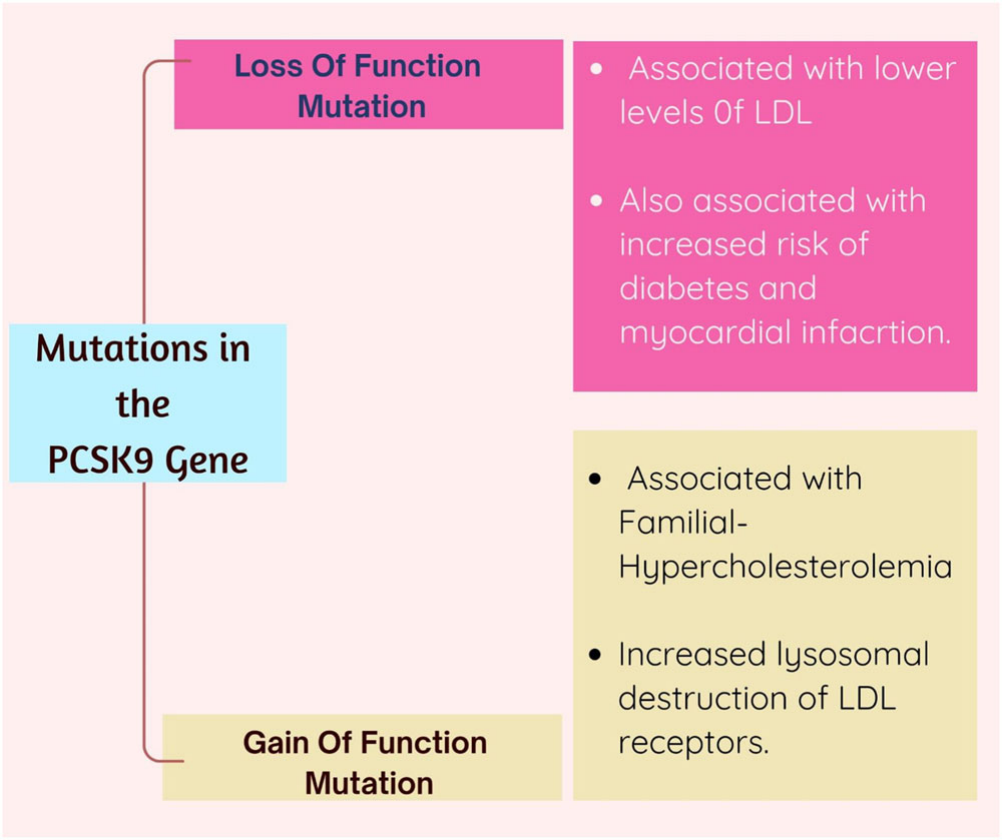
Genetic basis of PCSK9 inhibition.

A surprising finding from sequencing analysis was that people with gain‐of‐function mutations in PCSK9 had more LDL receptors destroyed, which increased LDL‐C levels and increased the risk of atherosclerosis [5]. In contrast, those individuals carrying PCSK9 loss‐of‐function variants had increased numbers of their LDL receptors recirculated back to the cell surface, promoting the clearance of LDL‐C from circulation and potentially protecting against atherosclerosis [6]. Even a 15% decrease in LDL‐C levels was linked to a significant 47% reduction in the risk of coronary artery disease (CAD), according to studies examining the incidence of CAD in people with PCSK9 loss‐of‐function variants [7]. These genetic discoveries opened the door for the creation of PCSK9 inhibitors, a novel class of treatments that aim to inhibit PCSK9 and boost LDL receptor activity, resulting in more effective LDL‐C clearance from the bloodstream. Since PCSK9 inhibitors have become clinically available, research into their potential as a ground‐breaking method of managing atherosclerotic cardiovascular disease (ASCVD) and lowering cardiovascular risk has gained traction [8]. The silver lining seen throughout the changing landscape of cardiovascular medicine as a step toward better patient outcomes and a new front in the battle against ASCVD is the knowledge of the genetic basis behind PCSK9 inhibition and its effects on LDL‐C levels. (Figure 2) summarizes the genetics and evolution of PCSK‐9 Inhibitors (see Figure 2).

Targeting low‐density LDL‐C, a major modifiable risk factor associated with ASCVD, PCSK9 inhibitors represent a major milestone in the treatment of ASCVD [9]. LDL‐C has been meticulously studied with several large prospective cohort studies, Mendelian randomization studies, and randomized clinical trials (RCTs), all of which have consistently shown the log‐linear relationship between LDL‐C and the risk of ASCVD [10]. Thus, statins have been commonly used and proven effective as LDL‐C‐lowering therapies. Despite their proven effectiveness, other therapeutic approaches need to be outlined, particularly in high‐risk patients in whom the desired reduction in LDL‐C levels with statins alone may not be found. On the other hand, a new class of cholesterol‐lowering therapies—PCSK9 inhibitors—have come into existence [1]. The drugs block PCSK9 and increase the number of liver surface LDL receptors, thereby improving the clearance of LDL from circulation [11]. The ultimate goal of this state‐of‐the‐art review article is to reassess PCSK9 inhibitors from an updated perspective following the recent completion of a series of clinical outcome trials. PCSK9 inhibitors hold great promise in cardiovascular medicine, either as adjunctive to statins for most patients or as monotherapy for the most appropriate candidates or patients at high cardiovascular risk [1, 2]. As the efficacy and safety of PCSK9 inhibitors can continue to be delineated, we can look forward to the evolution of these novel treatments to change the paradigm in the management of ASCVD and better outcomes for patients at high risk for cardiovascular events.

## 2 Decoding the Genetics of PCSK9: Unraveling the Impact on Cardiovascular Health

The genetic basis of PCSK9's role in cholesterol regulation was determined using linkage analysis in families with FH [2]. These families had high levels of LDL‐C, but no mutation could be detected in either of the FH‐causing genes. By using linkage analysis, a likely gene was found on chromosome 1p, which was shown to be the gene PCSK9, part of the proprotein convertase gene family [12]. Upon scrutiny of the DNA from patients with high cholesterol, researchers found rare specific changes in the PCSK9 gene (p.S127R, p.F216L, and p.D374Y). These changes were found only in high‐cholesterol patients and matched their phenotype perfectly [13]. This finding, in turn, identified PCSK9 as the third gene responsible for FH. When PCSK9 is overactive because of these particular changes in the gene, it is known as gain‐of‐function or GOF mutations, leading to the destruction of more LDL receptors. Therefore, more LDL‐C is available and the level in the blood is high. When PCSK9 is underactive because of these particular changes in the gene, it is described as having loss‐of‐function or LOF mutations [14]. There is less destruction of LDL receptors, and therefore, LDL‐C levels are lower (Figures 1 and 2).

Many reports have established an association of PCSK9 GOF mutations with ASCVD. It is challenging to conclusively link these mutations to an increased risk of ASCVD because they are so uncommon. A striking phenomenon is that some of those patients who have rare multiple combinations of gene changes, including both PCSK9 GOF and LDLR LOF, will have an LDL level between that of FH and normal cholesterol. Some GOF variant carriers possibly have milder phenotypes [15]. Contrarily, carriers of PCSK9 LOF variants generally have low LDL‐C levels and low ASCVD risk [16]. Genetic studies using Mendelian randomization to predict multiple populations have confirmed that individuals with a PCSK9 LOF variant have significantly reduced LDL‐C and ASCVD risk [17]. There is also a very interesting finding: people who have two copies of PCSK9 LOF variants (homozygotes) have an extremely low LDL‐C level without obvious health problems.

Genome‐wide analyses of common PCSK9 variants have suggested small but very consistent effects on LDL‐C levels. The rates of ASCVD events appear to roughly track the extent of LDL‐C lowering [18]. However, some suggestions are made that the PCSK9 LOF variants may be associated with higher blood sugar levels and an increased risk for type 2 diabetes [19]. Genetic advances in PCSK9 have led to the invention of PCSK9 inhibitors that became a novel, highly promising treatment for lowering LDL‐C levels and, ideally, decreasing the risk of ASCVD. These inhibitors were proven to be effective and safe in clinical trials and show much promise in the future of cardiology. Although PCSK9 inhibitors target a fundamental step in cholesterol regulation, current and future research deems it necessary to describe the general impact of such inhibitors on human health and disease, which may change the understanding of cholesterol regulation modulation dramatically in the context of heart health and other metabolic processes.

## 3 Clinical Significance of PCSK9 Inhibitors

PCSK9 is a major player in regulating body cholesterol and acts in the bloodstream to change the transport of cholesterol. If one thinks of the PCSK9 as an extra, less typical “key” to the LDL receptor (LDLRs), then it competes with more typical “keys” like LDL and other particles that carry lipids [20]. The paradox here is how a low‐concentration protein (100–300 ng/mL) of similar size to the LDL receptor regulates almost 70% of the billions of LDL receptors on the surface of a liver cell [21]. The interaction is not a simple one to one of PCSK9 and LDLR; their activities are amplified. Interestingly, while PCSK9 enters cells using LDLRs, most of it stays unchanged within cells for hours, suggesting a recycling process [22]. This will allow a single PCSK9 molecule to recycle to the cell surface and act on newly cycling LDL receptors. PCSK9 demonstrates an entirely different type of interaction than the traditional way ligands interact with receptors [23].

Half of PCSK9 in the bloodstream is bound to LDL or Lp(a) particles and the remainder circulates free or is associated with HDL. The PCSK9 bound to LDL is largely intact, and free PCSK9 is generally shorter and may have less affinity for LDLR [24]. Since there are more LDL particles than PCSK9, there is about one PCSK9‐containing LDL particle to every 500 naturally occurring LDL particles in the bloodstream [25]. This formulates the paradox that a few LDL particles carry the agent that is affecting LDL receptor function and thus, the overall cholesterol balance. This functions in concert with intricate cellular processes, including the SREBP and LXR pathways.

In terms of atherosclerosis (hardening of arteries), the effects of circulating PCSK9 are not completely clear. Analyzing clinical trials of PCSK9 inhibitors might not fully predict its impact [26]. PCSK9 can diffuse through the interstitial spaces of artery plaques, and the cells in the arterial plaques are capable of responding to the PCSK9. If the PCSK9 is acting normally, then how much LDLR is created by these cells should reflect the local PCSK9 concentration. Unlike in the liver, it is not healthy for cholesterol to be taken up efficiently into plaques since it might cause the plaque to grow or become vulnerable to rupture [27]. Blocking PCSK9 could increase LDLR on plaque cells and cause them to accumulate even more cholesterol in the blood. In relation to this, the direct effects of PCSK9 may also be inflammatory in the blood vessels, possibly helped by interactions with LDLR‐related protein 1 (LRP1) [28]. In the blood, PCSK9 inhibitors could have both favorable and unfavorable effects on atherosclerosis, and it is difficult to gain insight into the overall change, mainly because reducing plasma LDL‐C is a highly successful strategy.

**Figure 3:**
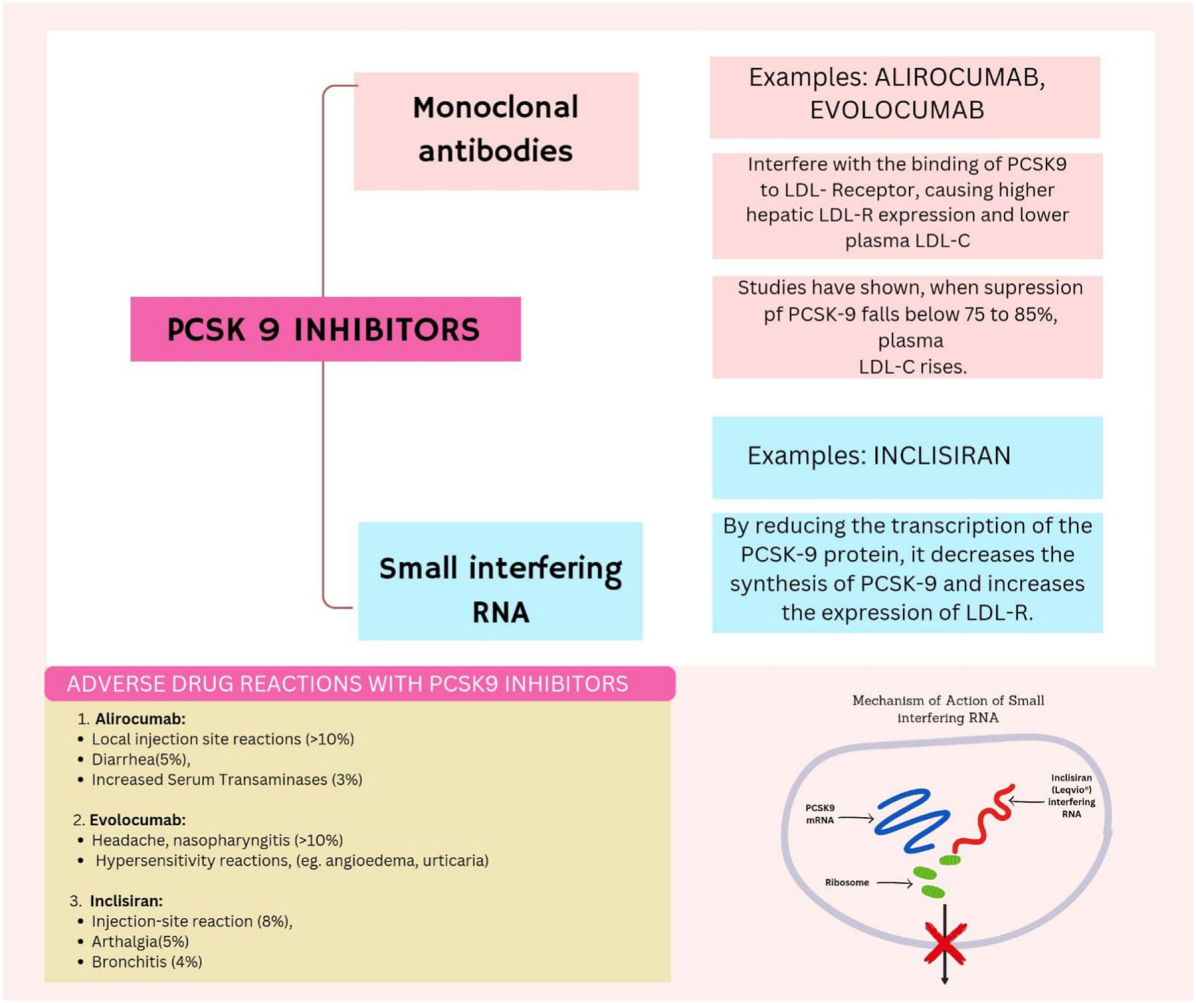
Mechanism of PCSK9 inhibition by monoclonal antibodies.

A potent way to attain a robust reduction in cholesterol is to block PCSK9 action in the blood via monoclonal antibodies (Figure 3). Alirocumab and Evolocumab are among these antibodies and they are engineered to combine with circulating PCSK9 in a 1:1 molar ratio [29]. By doing this, they release the bound PCSK9, thus preventing it from exerting its action on the LDL Receptors (LDLRs). There will, therefore, be more LDL receptors on the liver cell surface, hence more removal of the LDL particles from the blood and a robust reduction in plasma LDL‐C. Alirocumab (75–150 mg) or Evolocumab (140–420 mg) administered parenterally results in plenty of antibodies in comparison to the target levels of PCSK9 [30]. Within a few hours, these antibodies sequestered all circulating PCSK9 and continued to bind newly secreted PCSK9 for days. As a result, people receiving these antibodies have almost no free PCSK9, but the total PCSK9 levels go up a lot, sometimes even 20 times higher [31]. This big increase in PCSK9 has a few explanations. The antibodies slow down the clearance of PCSK9 compared to free PCSK9 because the antibodies cannot use the LDLR pathway. New PCSK9 produced after the injection also stays in circulation as immune complexes [32]. And blocking PCSK9's return to the liver might activate other pathways that increase PCSK9 production. Interestingly, the rise in cellular PCSK9 because of the antibodies is not really important [33].

A clinical trial called the SPECIAL study is investigating the safety and efficacy of the PCSK9 inhibitor evolocumab in patients with non‐ST segment elevation acute coronary syndrome (NSTE‐ACS) and multivessel CAD who have undergone culprit vessel revascularization [34]. The researchers are specifically evaluating patients with non‐culprit artery critical lesions, defined as stenosis degrees between 50% and 75%. This patient population was chosen because previous research has not determined if PCSK9 inhibitors would have similar plaque‐reversing effects in patients with this degree of stenosis. The SPECIAL study evaluates the effects of evolocumab on several parameters, including minimum FCT, lipid levels, and MACE. This study will provide more information about the potential benefits of PCSK9 inhibitors in this specific patient population [35].

Although PCSK9 requires liver‐derived VLDLs/triglycerides to be produced, the effect on serum triglyceride levels of PCSK9 inhibitors is relatively modest, likely due in large part to the fact that other triglyceride‐rich apoB containing lipoproteins may blunt PCSK9 inhibitor effects on triglycerides compared with LDL‐cholesterol [36]. This “secondary” sequestration of PCSK9, as part of the immune complex, helps in evaluating apparent resistance to PCSK9 inhibitors [37]. Because serum PCSK9 would typically be high if the antibodies were functional (i.e., reflecting true resistance), but low if PCSK9 was not inhibiting the LDLR, nearly half of PCSK9 is bound to LDL particles in the circulation [38]. This suggests that a large portion of the immune complexes generated by these injections of antibodies is likely LDL‐bound. The clinical importance of this is not yet known, but this may be a useful tool in finding out which LDL particles are carrying PCSK9 [39].

In addition to traditional lipid parameters, recent studies suggest that lipoprotein ratios, such as the ratio of total cholesterol to HDL‐cholesterol (TC/HDL‐C), LDL‐cholesterol to HDL‐cholesterol (LDL‐C/HDL‐C), triglycerides to HDL‐cholesterol (TG/HDL‐C), and non‐HDL‐cholesterol to HDL‐cholesterol (non‐HDL‐C/HDL‐C), may provide additional information about cardiovascular risk [40, 41]. The research found that South Asian patients with ACS had higher mean values for several lipoprotein ratios than Caucasian patients, even though the results were not statistically significant. Hence further research is needed to evaluate the cardiovascular risk in specific ethnic groups such as South Asians [42].

## 4 Pioneering Insights From Cardiovascular Trials: Unveiling the Clinical Impact of PCSK9 Inhibitors

**Figure 4:**
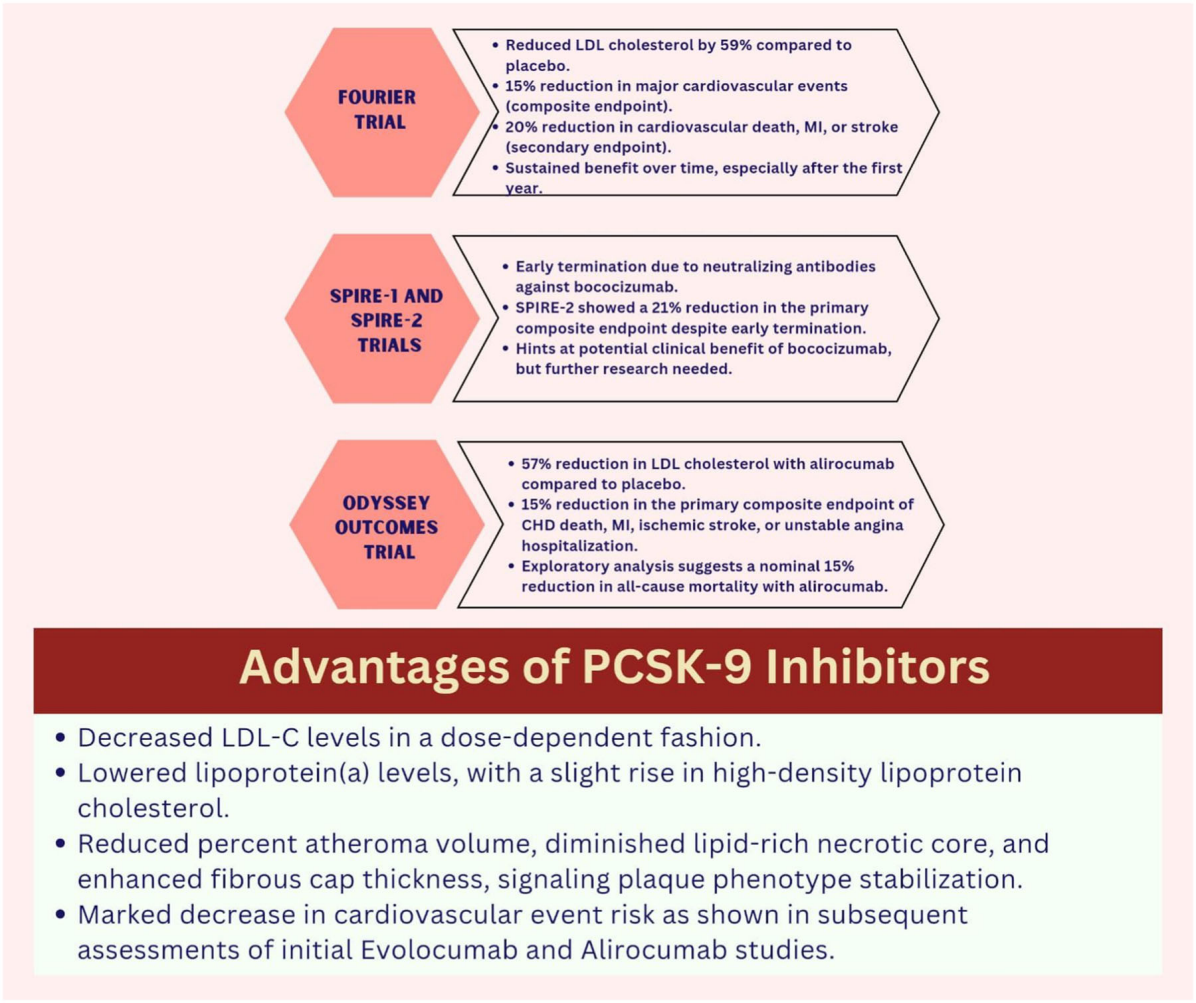
Summary of major cardiovascular trials on PCSK9 inhibitors.

Cardiovascular trials have played a crucial role in advancing our understanding of PCSK9 inhibitors, a novel class of medications, by providing valuable insights into their efficacy and safety. Three major trials— FOURIER, SPIRE‐1 and SPIRE‐2, and the ODYSSEY Outcomes trial—have provided significant evidence for the use of PCSK9 inhibitors in managing cardiovascular diseases (Figure 4).

### 4.1 Fourier Trial

The FOURIER trial stood as a pivotal cardiovascular outcome trial that aimed to assess the effectiveness and safety of evolocumab, a PCSK9 inhibitor. This trial enrolled a substantial cohort of 27,564 patients who exhibited clinically evident ASCVD and had a history of myocardial infarction (MI), non‐hemorrhagic stroke, or symptomatic peripheral artery disease (PAD). The study subjects were already undergoing optimized lipid‐lowering therapy, primarily high‐intensity statins, and presented baseline LDL‐C levels of 70 mg/dL or higher, or non‐HDL cholesterol levels of 100 mg/dL or higher.

Participants were randomized to receive either subcutaneous Evolocumab injections (140 mg every 2 weeks or 420 mg monthly) or matching placebo injections. The primary efficacy endpoint of the study was the composite of major cardiovascular events, encompassing cardiovascular death, MI, stroke, coronary revascularization, or hospitalization for unstable angina. The primary secondary endpoint was the composite of cardiovascular death, MI, or stroke.

The trial results demonstrated that Evolocumab notably reduced LDL‐C levels by 59% at 48 weeks compared to the placebo, resulting in a substantial mean absolute reduction of 56 mg/dL to a median level of 30 mg/dL. Importantly, the trial exhibited a 15% reduction in the risk of the primary endpoint (HR 0.85, 95% CI 0.79–0.92) and a 20% reduction in the main secondary endpoint (HR 0.80, 95% CI 0.73–0.88) with Evolocumab treatment. The trial's findings also indicated that the reduction in cardiovascular events became more pronounced after the initial year of treatment, suggesting a sustained clinical benefit over time [43].

However, a reanalysis based on regulatory data revealed some inconsistencies between the information in the clinical study report (CSR) and that in the 2017 primary trial results publication. For 360 out of 870 deaths (41.4%), the cause of death adjudicated by the FOURIER clinical events committee differed from that declared by the local clinical investigator. After readjudication, it was noted that cardiac deaths were numerically, but non‐significantly, higher in the Evolocumab group (113) than in the placebo group (88; relative risk (RR) 1.28, 95% CI 0.97–1.69, *p* = 0.078), whereas noncardiac vascular deaths were similar between groups (37 in each; RR 1.00, 95% CI 0.63–1.58, *p* = 0.999). The reported HR for cardiovascular mortality in the original trial analysis was 1.05 (95% CI 0.88–1.25); after readjudication, a greater (although still nonsignificant) relative increase in cardiovascular mortality in the Evolocumab treatment group was found (RR 1.20, 95% CI 0.95–1.51, *p* = 0.13) [44].

### 4.2 SPIRE‐1 and SPIRE‐2 Trials

The SPIRE‐1 and SPIRE‐2 trials investigated the PCSK9 inhibitor Bococizumab in a large cohort of 27,438 patients with a history of cardiovascular disease or diabetes. The trials were designed to evaluate Bococizumab's effects on LDL‐C levels and cardiovascular outcomes. However, both trials were terminated prematurely due to the development of neutralizing antibodies against Bococizumab. These antibodies diminished the drug's ability to lower LDL‐C levels over time, ultimately leading to a waning of its therapeutic effect. Despite the early termination, the SPIRE‐2 trial demonstrated a notable finding: a 21% risk reduction in the primary composite endpoint in the Bococizumab group compared to the placebo group. The primary composite endpoint included cardiovascular death, nonfatal MI, or nonfatal stroke. This suggests that Bococizumab may have provided a clinical benefit. This highlights the importance of monitoring for neutralizing antibodies in patients receiving PCSK9 inhibitors and underscores the need for continued research into strategies to mitigate this challenge [45, 46].

### 4.3 Odyssey Outcomes Trial

The ODYSSEY outcomes trial centered around investigating the effects of alirocumab, another PCSK9 inhibitor, in patients who had experienced a recent acute coronary syndrome (ACS), such as MI or unstable angina, within 1–12 months before enrollment. The trial enrolled 18,924 patients who were already on high‐intensity statin therapy. Alirocumab treatment resulted in a significant 57% reduction in LDL‐C levels compared to the placebo. The primary composite endpoint, which included coronary heart disease (CHD) death, MI, ischemic stroke, or hospitalization for unstable angina, exhibited a 15% reduction with alirocumab compared to the placebo (HR 0.85, 95% CI 0.78–0.93, *p* = 0.003). Additionally, an exploratory analysis hinted at a nominal 15% reduction in all‐cause mortality with alirocumab, although statistical significance was not achieved [47].

Overall, these pivotal cardiovascular trials have illuminated the potential of PCSK9 inhibitors as effective tools in managing cardiovascular disease, particularly in high‐risk patient populations. The insights gained from these trials highlight the clinical relevance of PCSK9 inhibitors in reducing cardiovascular events, improving lipid profiles, and potentially enhancing overall patient outcomes.

## 5 Additional Clinical Considerations

Elevated Lipoprotein(a) is an independent risk factor for cardiovascular disease and has been linked to a higher risk for coronary, peripheral artery, and cerebrovascular disease. PCSK9 inhibitors not only effectively lower low‐density lipoprotein cholesterol (LDL‐C) levels but can also reduce lipoprotein(a) concentrations by 20%–25% [40, 41]. An analysis of the ODYSSEY Outcomes trial demonstrated that patients with recent ACS and LDL‐C levels near 70 mg/dL on optimized statin treatment who also had elevated lipoprotein(a) levels experienced a significant reduction in major adverse cardiovascular events (MACE) with the addition of the PCSK9 inhibitor alirocumab [47]. These findings suggest that measuring lipoprotein(a) levels in patients with recent ACS and LDL‐C levels near 70 mg/dL, who are already receiving optimal statin therapy, could help identify individuals who might derive a greater clinical benefit from PCSK9 inhibitor treatment.

Elevated levels of LDL‐C are the hallmark of a typical autosomal genetic disorder named FH. Based on various estimations, the prevalence of the disease is about 1 in 250 heterozygotes and 1 in 300,000 homozygotes. A mutation leading to inactivation in a number of genes that are involved in the LDL receptor (LDLR) pathway or its related proteins, which are key elements, leads to FH. In addition, a subset of patients can be accounted for by polygenic factors.

Clinical suspicion for heterozygous FH develops when adult patients show concomitant clinical indicators and an LDL‐C level that exceeds the cutoff of 190 mg/dL. Before the development of proprotein convertase subtilisin/kexin type 9 (PCSK9) inhibitors, the therapeutic strategy historically relied on statins as the main intervention. To achieve the best LDL‐C management, certain cases may have required the addition of alternative oral nonstatin agents. Extracorporeal apheresis targeting LDL particles became routine after people who are homozygous for FH showed little response to conventional therapeutic modalities.

It is noteworthy that the FDA has already approved the usage of the drugs Alirocumab and Evolocumab in managing the disorder. Thorough clinical studies have proven that the simultaneous administration of these PCSK9 inhibitors with standard therapies significantly lowers LDL‐C levels in FH patients. Within this context, a substantial proportion—ranging from 60% to 80%—of FH patients have successfully attained the stringent guideline‐endorsed LDL‐C targets upon treatment with PCSK9 inhibitors, showcasing a remarkable improvement compared to the meager achievement rate of less than 5% among placebo recipients.

The clinical significance of PCSK9 inhibitors had surpassed classic LDLR genetic defects. Of importance, FH patients with genetic mutations in APOB or PCSK9 have responded well to PCSK9 inhibition therapeutically. The initial reports also indicate that the benefits of PCSK9 inhibition might be extended to patients with autosomal recessive hypercholesterolemia.

As a result of the perceived discrepancy between the prevalence of patient‐reported SAMS in real‐world situations and the relatively low incidence of adverse muscular events documented in RCTs, the use of PCSK9 inhibitors has been extended to include patients suffering from statin‐associated muscle symptoms (SAMS). Accurately attributing reported symptoms to statin use and putting into practice therapeutic strategies in line with RCT results and cholesterol management guidelines are necessary to address the difficulties associated with SAMS research in clinical trials.

In summary, FH is a disorder characterized by high LDL‐cholesterol levels and is considered one of the most interesting and provocative in both research and practice. PCSK9 inhibitors, Alirocumab and Evolocumab, have changed the face of therapy in FH patients with an opportunity for successful reduction in LDL‐cholesterol levels according to guidelines (Figure 3). These agents have been effective not only in patients with mutations in the classical form of the LDLR gene but also in those with alternative genetic causes and those suffering from SAMS. Research will continue to identify the whole potential of inhibiting PCSK9 usage to alleviate the burden of FH and its complications.

## 6 Advancements in PCSK9 Inhibition

A major example of one of these novel approaches in the expanding field of therapeutic strategies for the inhibition of proprotein convertase subtilisin/kexin type 9 (PCSK9) is the use of small interfering RNAs (siRNAs). A prominent exemplar of this approach is Inclisiran, a siRNA‐based drug characterized in lipid management by unique features.

Inclisiran is a chemically synthesized siRNA conjugated with a ligand having three N‐acetyl‐galactosamine (GalNAc) residues. The presence of GalNAc moieties greatly enhances the interactions between Inclisiran and the asialoglycoprotein receptor, which is highly expressed on hepatocytes and mediates, therefore, the high degree of specificity associated with Inclisiran in the liver cells. Hence, the targeting capabilities are highly important for the proper intracellular delivery of the therapeutic agent [48].

Once inside hepatocytes, Inclisiran enters the natural RNA interference pathway and makes a crucial interaction with the RNA‐induced silencing complex (RISC). Through this molecular mechanism, the RISC complex is permitted to cleave PCSK9 mRNA selectively, leading to a reduction in the synthesis of PCSK9. The lower number of available PCSK9 leads to more active LDL receptors, hence increasing the clearance of LDL‐C from circulation. A standout feature of Inclisiran is its long‐lasting effect, where a single administration can lower LDL‐C for several months, eliminating the need for frequent dosing. This extended duration of action offers a significant logistical advantage over PCSK9 monoclonal antibodies, which require more regular administration [48].

Clinical findings from the phase II ORION‐1 trial have unmasked the therapeutic promise of Inclisiran. Administration of 300 mg of the drug every 6 months led to an average reduction of LDL‐C by approximately 50%. Notably, this activity was conducted with a favorable safety profile as there was no elevated rate of serious adverse events. Injection site reactions, common with injectable drugs, developed in only 7% of patients in the 300 mg cohort. The trajectory of promise of Inclisiran is, perhaps, best epitomized by the phase III trials that continue to be designed to further prove its safety and efficacy. A particularly salient example is that of the HPS‐4/TIMI 65–ORION‐4 trial that currently spans 15,000 patients and is planned to last a total of 5 years. Medication is administered on Days 1, 90, and then every 6 months. This dedicated trial of cardiovascular outcomes will provide invaluable insights into the long‐term impact of Inclisiran on clinical cardiovascular endpoints [49].

The concept of “life‐long cholesterol burden” is applicable when considering early intervention strategies. Effective control of circulating LDL‐C by an intervention such as Inclisiran may help reduce the burden on ASCVD. This will be cumulative and hence affect one's ASCVD risk over one's lifetime. Particularly, in those with some predisposition to having higher circulating levels of LDL‐C, the development of atherosclerotic manifestations, such as in those with FH, early in life, is likely to increase dramatically. The hypothesis of initiating LDL‐C‐lowering interventions, such as Inclisiran, from an early age, even as early as 25, introduces the tantalizing prospect of significantly postponing the clinical onset of ASCVD.

The horizon of PCSK9 inhibition is broadening with researchers' exploration of novel strategies, including immunization with PCSK9‐mimicking peptides, gene editing through CRISPR‐Cas9 technology, and interventions to achieve and maintain enduringly low plasma LDL‐C levels. Yet, these impressive strategies are still in the experimental phase; their possible translation into efficient and safe intervention would revolutionize the management of cardiovascular disease. Interestingly, these strategies may not only effectively lower LDL but may also confer long‐lasting protection against atherosclerosis, shifting the paradigm from treating advanced vascular disease to anticipating the onset of such a disease—a concept with truly transformational implications to the field of cardiology.

## 7 Safety and Tolerability of PCSK9 Inhibitors

PCSK9 inhibitors are generally well‐tolerated, more research is needed to fully understand their long‐term safety profile, particularly in comparison to statins. Statin therapy, a common treatment to lower LDL‐C, can reduce ClC‐1 expression in skeletal muscle, which may lead to muscle‐related side effects [50, 51, 52]. This finding suggests that measuring ClC‐1 expression might help identify patients at a higher risk of experiencing statin‐induced myopathy. PCSK9 inhibitors may be a useful treatment option for patients who experience SAMS and cannot tolerate statins. The most common side effects associated with PCSK9 inhibitors are injection site reactions, which are usually mild and transient. Some studies have raised concerns about a potential link between PCSK9 inhibitors and an increased risk of new‐onset diabetes. However, the evidence is inconclusive, and further research is needed to clarify this association. A large clinical trial, called the ODYSSEY Outcomes trial, examined the safety and efficacy of the PCSK9 inhibitor alirocumab in patients with a recent ACS [47]. This trial found that alirocumab was generally safe and well‐tolerated, with a safety profile comparable to that of the placebo group. Researchers are currently conducting a clinical trial, called the SPECIAL study, to investigate the safety and efficacy of the PCSK9 inhibitor Evolocumab in patients with non‐ST segment elevation ACS and multivessel CAD, who have undergone culprit vessel revascularization. This trial is specifically evaluating patients with critical lesions (stenosis degree between 50% and 75%) in non‐culprit arteries.

## 8 Conclusion

In summary, PCSK9 inhibitors represent a novel class of pharmacological agents that has revolutionized and dramatically changed the landscape in treating elevated LDL cholesterol and cardiovascular diseases. This new class, led by agents like Alirocumab and Evolocumab, is made up of fully human IgG antibodies that selectively bind to circulating PCSK9. In this manner, the inhibitors are able to reduce the binding affinity of PCSK9 to the LDL receptor, in an orchestrated cascade marked by the great reduction of LDL cholesterol levels.

As a result, this cascade engenders an accelerated rate of clearance with respect to LDL particles, in the process causing significant and marked reductions in plasma LDL‐C levels. These developments offer a propitious and encouraging therapeutic modality, particularly for individuals afflicted with FH or those with clinically evident ASCVD.

While the potential of PCSK9 inhibitors is undoubtedly promising, it remains incumbent upon the scientific community to pursue ongoing investigations and meticulously designed clinical trials. These efforts are necessary to truly understand its complex tapestry over long‐term safety and sustained effectiveness. For example, future studies could investigate the potential role and clinical utility of incorporating lipoprotein ratios, such as TC/HDL‐C, LDL‐C/HDL‐C, TG/HDL‐C, and non‐HDL‐C/HDL‐C, along with lipoprotein(a) measurements, into the assessment of patients being considered for PCSK9 inhibitor therapy. Further research, like the SPECIAL study, is needed to refine PCSK9 inhibitor treatment strategies, especially for patients who gain the most benefit, such as those intolerant to statins. It is notable that the horizon of PCSK9 inhibition is further illuminated by the potential interplay of complementary therapeutic strategies and the continuous evolution of gene editing technologies. As these avenues unfurl, the future of PCSK9 inhibition holds the promise of a remarkable transformation in cardiovascular medicine.

## Author Contributions

**Bijay Mukesh Jeswani:** conceptualization; writing–original draft; writing–review and editing. **Shubhangi Sharma:** writing–original draft; writing–review and editing. **Sawai Singh Rathore:** writing–original draft; writing–review and editing. **Abubakar Nazir:** writing–original draft; writing–review and editing. **Rohit Bhatheja:** writing–original draft; writing–review and editing. **Kapil Kapoor:** writing–original draft; writing–review and editing.

## Disclosure

The lead author Abubakar Nazir affirms that this manuscript is an honest, accurate, and transparent account of the study being reported; that no important aspects of the study have been omitted; and that any discrepancies from the study as planned (and, if relevant, registered) have been explained.

## Data Availability

The data that support the findings of this study are available from the corresponding author upon reasonable request.
